# The double-edged sword of evolution

**DOI:** 10.7554/eLife.29056

**Published:** 2017-07-03

**Authors:** Etty Kruzel-Davila, Karl Skorecki

**Affiliations:** 1 Department of Nephrology, Rambam Health Care Campus, Haifa, Israel; 2 Department of Medical and Research Development, Rambam Heath Care Campus, Haifa, Israelk_skorecki@rambam.health.gov.il; 3 Rappaport Faculty of Medicine, Technion – Israel Institute of Technology, Haifa, Israel

**Keywords:** Trypanosoma brucei, chronic kidney disease, Human African Trypanosomiasis, sleeping sickness, Trypanosoma brucei gambiense, Trypanosoma brucei rhodesiense, Human

## Abstract

Two gene variants provide different levels of protection against sleeping sickness, but this comes with an increased risk of developing chronic kidney disease.

**Related research article** Cooper A, Ilboudo H, Alibu VP, Ravel S, Enyaru J, Weir W, Noyes H, Capewell P, Camara M, Milet J, Jamonneau V, Camara O, Matovu E, Bucheton B, MacLeod A. 2017. *APOL1* renal risk variants have contrasting resistance and susceptibility associations with African trypanosomiasis. *eLife* **6**:e25461. doi: 10.7554/eLife.25461

African trypanosomiasis is a disease caused by trypanosome parasites that affects humans and other animals in sub-Saharan Africa. In humans, the disease manifests itself as sleeping sickness, and it can be fatal if untreated. Previous research has shown that a protein called APOL1 (short for Apolipoprotein 1) protects against many different species of trypanosome parasites in humans (Vanhamme et al., 2003). However, two subspecies have evolved independent mechanisms to become resistant to APOL1: *Trypanosoma brucei rhodesiense*, which predominates in East Africa, and *Trypanosoma brucei gambiense*, which is more common in West Africa (Uzureau et al., 2013; Pays et al., 2014).

Consequently, in an evolutionary arms race between humans and these two subspecies of the parasites, two mutations of the *APOL1* gene, named G1 and G2 (G0 being the ancestral *APOL1* gene without mutations), have become more common in people in sub-Saharan Africa in the last 10,000 years. These mutations have previously been linked to a higher risk of developing chronic kidney disease in African-Americans (Genovese et al., 2010; Tzur et al., 2010). Laboratory studies have revealed that the blood of people who carry either of these variants, and the G2 variant in particular, is able to kill the *T. b. rhodesiense* parasites that are mostly found in East Africa (Genovese et al., 2010; Thomson et al., 2014). However, until now, it has not been clear if these variants provide protection outside the lab. Furthermore, it was not known why G1 variants are generally more common than G2 variants, and why the geographic distribution of G1 does not match the geographic distribution of *T. b. rhodesiense* in East Africa.

Now, in eLife, Annette MacLeod, Bruno Bucheton and colleagues – including Anneli Cooper of the University of Glasgow and Hamidou Ilboudo of CIRDES in Burkina Faso as joint first authors – report new insights into these questions from an evolutionary point of view (Cooper et al., 2017). Their results confirm that, as Theodosius Dobzhansky once said, "nothing in biology makes sense except in the light of evolution".

In two case-control studies, Cooper et al. examined if the two variants influenced how susceptible individuals were to African trypanosomiasis. In the first study, Cooper et al. showed that in a region of Uganda, which is in East Africa, individuals with G2 were protected against *T. b. rhodesiense*, and were five times less likely to get the disease than people with G0 or G1.

In the second study, which was conducted in Guinea in West Africa, gene samples were obtained from three different groups: non-infected controls; individuals with a latent infection; and individuals with active symptomatic sleeping sickness. Neither gene variant could protect individuals against infection by *T. b. gambiense*. However, infected individuals carrying G1 were more likely to have a latent asymptomatic infection without parasites in their blood, whereas people carrying G2 were more likely to suffer from an active infection and to be severely unwell.

These results support the theory that the increase in G1 and G2 variants is linked to their protective effect against sleeping sickness, despite their deleterious effect on kidney health. In line with results of the laboratory studies (Genovese et al., 2010), the protective association of G2 against East African sleeping sickness caused by *T. b. rhodesiense* seems straightforward and consistent with its geographic distribution.

The protective properties of G1 against active sleeping sickness in people infected with *T. b. gambiense* seems to explain why this variant is so common amongst people in West Africa. On the other hand, the increased risk that people with G2 mutations face when infected with this parasite may explain why G2 is generally less common than G1, even if G2 is more efficient in killing *T. b. rhodesiense* parasites. Therefore, while the effects of evolutionary selection are strong, they are also more nuanced than previously appreciated (Figure 1).

**Figure 1.**
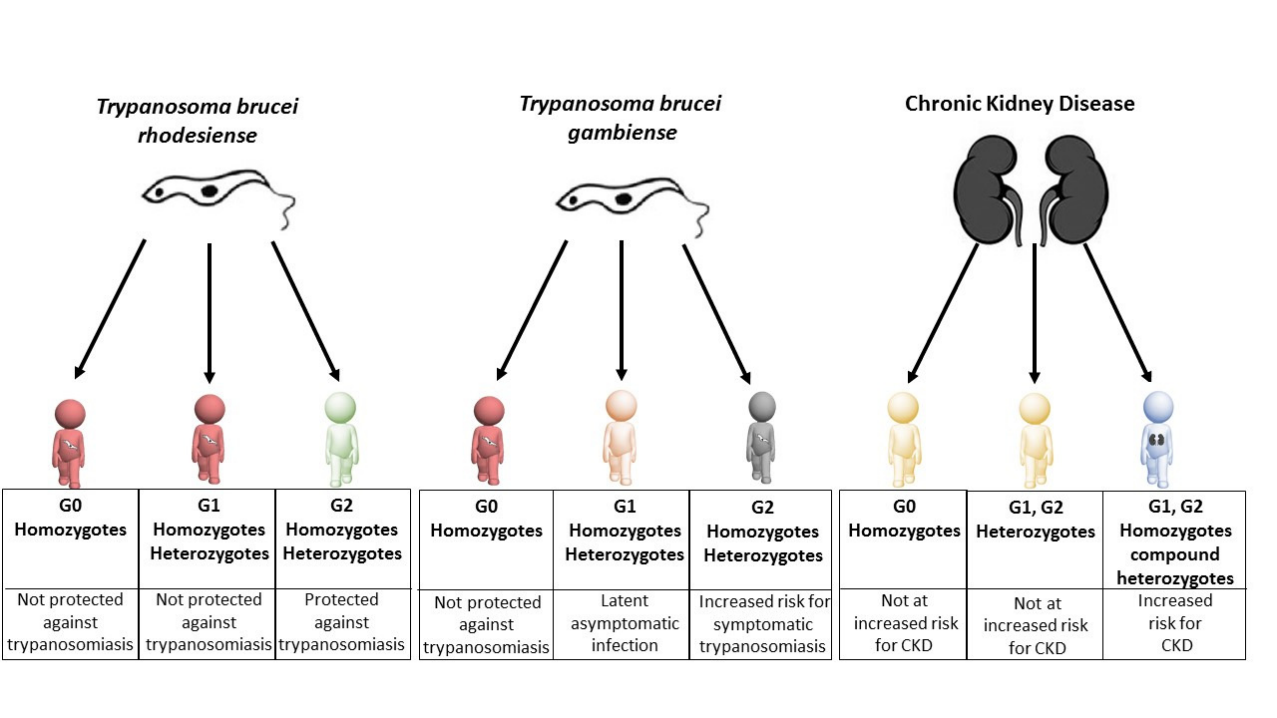
Individuals carrying different variants of the *APOL1* gene are protected against sleeping sickness and kidney disease to different extents. Individuals with two copies of wild-type *APOL1* (G0 homozygotes) are not protected against sleeping sickness caused by *T. b. rhodesiense* (red figure, left column) or *T. b. gambiense* (red figure, middle column), but they do not have an increased risk of chronic kidney disease - CKD (yellow figure, right column). Individuals with one copy of wild-type *APOL1* and one copy of the G1 variant (G1 heterozygotes), and individuals with two copies of the G1 variant (G1 homozygotes) are not protected against sleeping sickness caused by *T. b. rhodesiense* (second red figure, left column) and are more likely to have latent asymptomatic infection by *T. b. gambiense* (pink figure, middle column). Individuals with one copy of wild-type *APOL1* and one copy of the G2 variant (G2 heterozygotes), and individuals with two copies of the G2 variant (G2 homozygotes) are protected against sleeping sickness caused by *T. b. rhodesiense* (green figure, left column) but are at increased risk of developing symptomatic infection by *T. b. gambiense* (grey figure, middle column). Like G0 homozygotes, G1 heterozygotes and G2 heterozygotes do not have an increased risk of chronic kidney disease (second yellow figure, right column). However, G1 homozygotes, G2 homozygotes and compound heterozygotes (individuals with both G1 and G2) all have an increased risk of chronic kidney disease (blue figure, right column).

Despite these new insights, several pieces of the puzzle are still missing. For example, it is not clear how individuals infected by *T. b. gambiense* are protected from developing symptomatic infection. Changes to the innate immune system that affect key mediators of the inflammatory response could provide one explanation (Bucheton et al., 2011; Ilboudo et al., 2014). Cooper et al. suggest that G1 could potentially contribute to this resistance, but further research is needed to confirm this hypothesis.

Likewise, we do not understand why individuals with G2 have an increased risk of developing active symptomatic sleeping sickness caused by *T. b. gambiense, *nor why G2 is fairly common in West Africa, despite this risk. One explanation for this could be that G2 may protect against other pathogens. Lastly, the significant protective differences between G1 and G2 uncovered in this study raise the question of whether the two gene variants may also play different roles in chronic kidney disease.

The study by Cooper et al. highlights the contrasting protective effects of G1 and G2 against *T. b. gambiense* and *T. b. rhodesiense*. Their findings explain the specific evolutionary pressure that has led to an increase of the gene variants in West and East Africa, respectively. Immediate challenges are to unravel the underlying mechanisms that cause the differences in G1 and G2, to explore whether these differences may be translated to kidney disease risk mechanisms and triggers, and to find out if any other pathogens are involved in this intriguing evolutionary arms race.
